# Complete mitochondrial genome of *Idioscopus nitidulus* (Hemiptera: Cicadellidae)

**DOI:** 10.1080/23802359.2018.1437798

**Published:** 2018-02-10

**Authors:** Jaipal S. Choudhary, Naiyar Naaz, Bikash Das, Bhagwati P. Bhatt, Chandra S. Prabhakar

**Affiliations:** a ICAR Research Complex for Eastern Region, Research Centre, Ranchi, Jharkhand, India;; b ICAR Research Complex for Eastern Region, ICAR Parisar, P.O. Bihar Veterinary College, Patna, Bihar, India;; c Department of Entomology, Veer Kunwar Singh College of Agriculture (Bihar Agricultural University, Sabour), Dumraon, Bihar, India

**Keywords:** Mitogenome, mango pest, Auchenorrhyncha, insect, leafhopper

## Abstract

The complete mitogenome of *Idioscopus nitidulus* (Cicadellidae) was sequenced. It comprises 15,287 base pairs (bp), including 13 protein-coding genes (PCGs), 2 rRNA genes, 22 tRNA genes and a control region. The phylogenetic analyses based on concatenated thirteen protein-coding genes of mitogenomes recover the monophyly of Auchenorrhyncha (Fulgoromorpha + Cicadomorpha) and Sternorrhyncha as a sister group to Auchenorrhyncha. The complete mitogenome sequence of *Idioscopus nitidulus*is available in the GenBank with accession number: KR024406.

*Idioscopus nitidulus* (Walker) (Cicadellidae: Hemiptera) is one of the most serious and endemic species of mango leafhopper in India. Nymph and adults of *I. nitidulus* found to suck sap from young shoots, tender leaves and inflorescence resulting in 20–100% loss of inflorescence (Sohi and Sohi 1990).

In the present work, we sequenced the complete mitogenome sequence of *I. nitidulus* and compared it with mitogenome of other leafhopper species (*Empoasca vitis* and *Homalodisca coagulata*). The phylogenetic relationships among hemipteran insects based on the complete mitogenome sequences were also analysed.

Specimens of *I. nitidulus* were collected from mango tree at ICAR Research Complex for Eastern Region, Research Centre (ICAR RCER-RC), Ranchi, India (23° 45′ N latitude, 85° 30′ E longitude, elevation 620 m AMSL). The voucher specimens (ID: IN001) of the species are kept in the collection of ICAR RCER-RC, Ranchi, India. Adult *I. nitidulus* was subjected for mitochondrial DNA extraction using BioVision Mitochondrial DNA Isolation Kit according to manufacturer’s instructions (BioVision, Inc., Milpitas, CA). Illumina library preparation and sequencing was executed at the *Xcelris Labs Limited,* India. A contig identified as mitogenome was annotated with the MITOS web server (Bernt et al. 2012) applying the invertebrate mitochondrial genetic code. Phylogenetic analysis was carried out on the basis of 37 available mitogenomes of Hemipteran insects in GenBank including newly sequenced mitogenome of *I. nitidulus* with Maximum Likelihood (ML) methods using MEGA ver 6.0 (Tamura et al. 2013).

The complete mitogenome of *I. nitidulus* is a closed-circular double-stranded molecule of 15,287 bp in length and arrangements similar to two Cicadellidae mitogenomes available in GenBank (*E. vitis* and *H. coagulata*). It presents typical gene content and arrangement of other insect mitogenomes, i.e. 13 PCGs, 22 tRNA genes and 2 genes for rRNA subunits.

Phylogentic studies of *I. nitidulus* with other species of six superfamilies represented in our dataset are monophyletic. *I. nitidulus* was observed closer to *H. coagulata* followed by *E. vitis* phylogenetically among studied species of Hemiptera (Figure 1). The relationships recovered among the six planthopper families analysed here were consistent with the phylogeny of Fulgoroidea as reconstructed by Urban and Cryan (2007). This phylogenetic analysis added to current knowledge on the hemipteran phylogeny inferred from mitogenomes.

**Figure 1.**
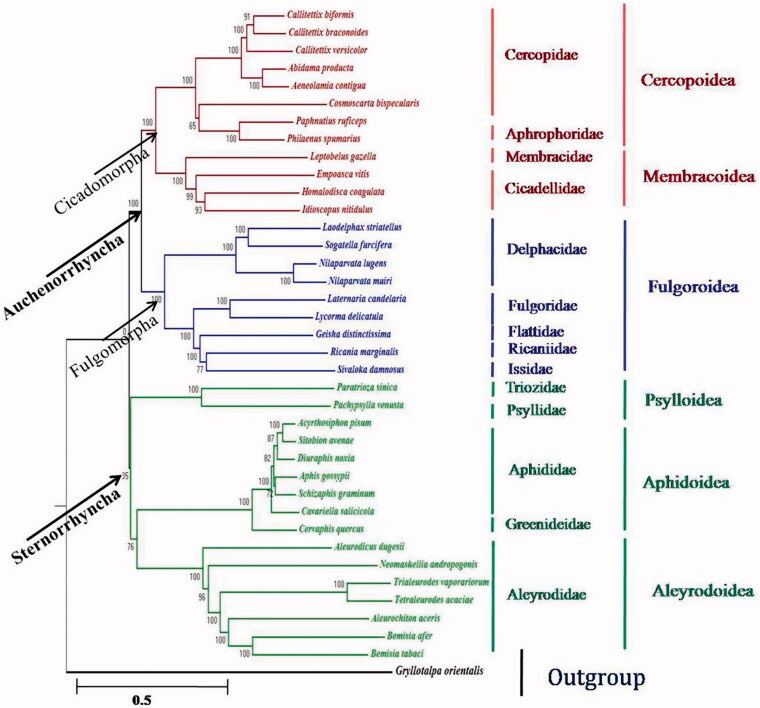
Maximum likelihood phylogenetic relationship inferred from the mitogenomes of 37 hemipteran insects. Tree based on 13 complete protein-coding genes from complete mitochondrial genome sequences.
